# Supplementary material for: Microstructure and Hydrophobicity of the External Surface of a Sonoran Desert Beetle
Source: Biomimetics (Basel). 2022 Mar 31;7(2):38. doi: 10.3390/biomimetics7020038 (PMC9036228; doi:10.3390/biomimetics7020038)
Supplement: Supplementary file 1 [file biomimetics-07-00038-s001.zip › biomimetics-1646099-supplementary.pdf]

Supplementary material

In this section, we provide supporting visual material that complements information provided in the main text.

In the first image shown below (Figure S1), the main natural mechanism that Namibian Desert Beetles use to collect water is schematized.

As described before, the use of this tilting angle position allows the beetle to harvest micro droplets of aerial moisture passively and by using the elytral heterogeneal hydrophobicity/hydrophilicity.

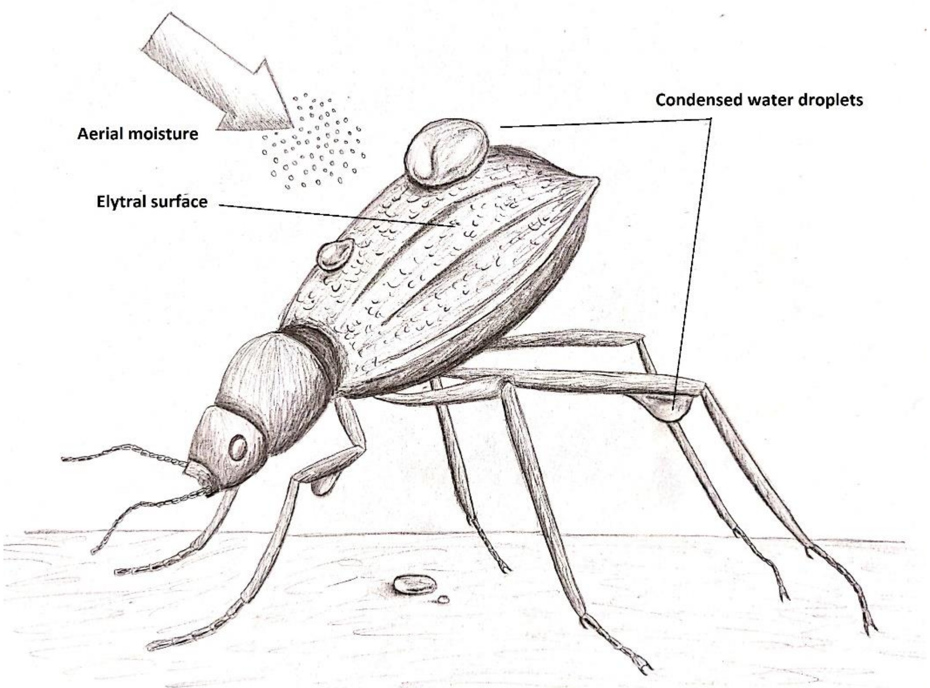

Figure S1: Head standing position as a putative mechanism for collecting atmospheric water in the elytra of beetles of the Namibian and Sonoran deserts (genera *Stenocara* and *Eleodes*, respectively, Tenebrionidae).
(Original drawing modified from https://asknature.org/strategy/water-vapor-harvesting/).

To improve fog collection, environmental conditions must be optimal for better harvesting. This depends on the aerial moisture available, depending on weather factors such as annual precipitation and temperature and also relative humidity. In figure S2 we present weather information for the two sampling sites. The informative data from Figure S2 was collected from Servicio Meteorológico Nacional (SMN).

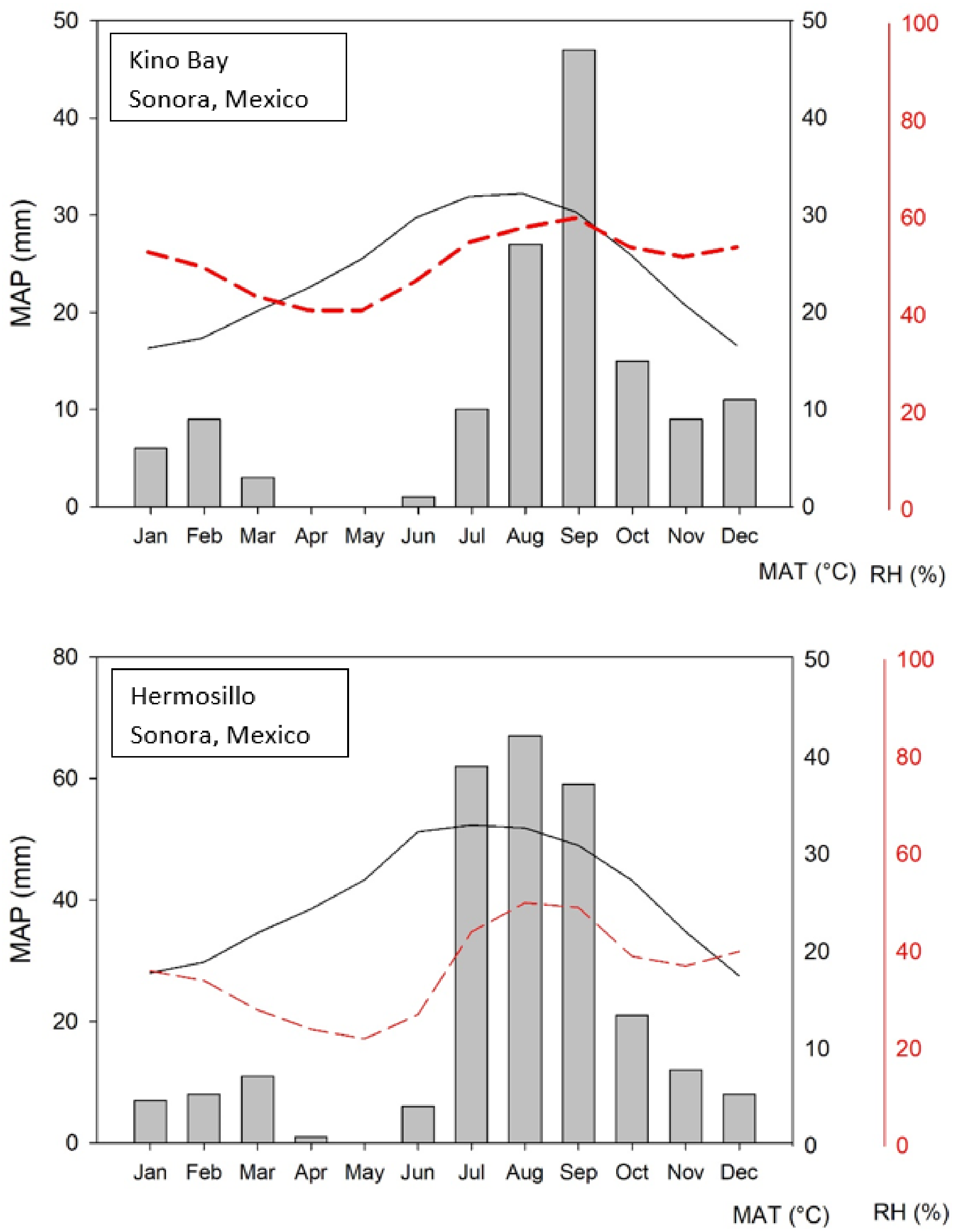

Figure S2. Monthly variation in mean precipitation (MAP, in mm), mean temperature (MAT, in °C, black line), and relative humidity (RH, in %; red, dotted line) along the year for both sampling sites. Note the difference in data range for RH.

Pitfall traps were installed in both sampling sites. In Figure S3 we present some of these traps.

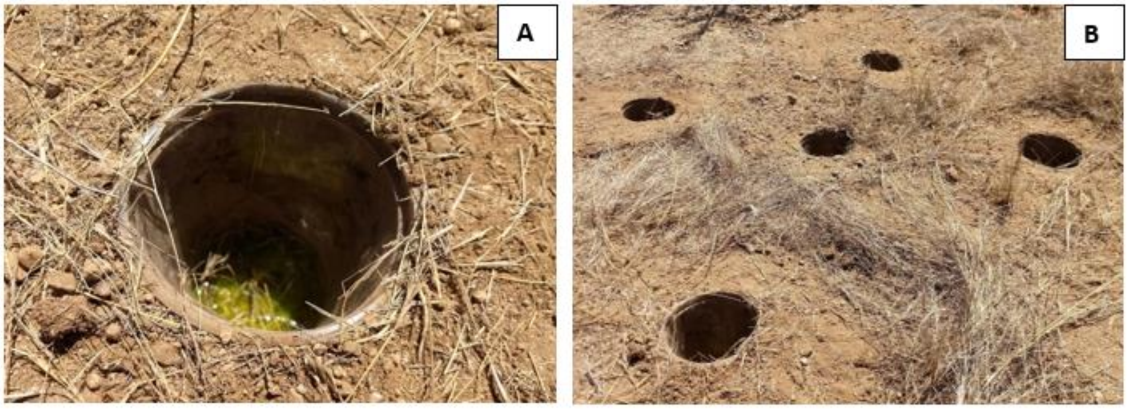

Figure S3. View of an individual pitfall trap (A) showing the antifreeze fluid used to preserve captured insects. View of a group of 5 pitfall traps (B) located along the transect.

Once the specimens were collected into the pitfall traps, about a week later, they were morphologically analyzed. This analysis consisted in the dissection of every individual´s elytra with a surgical blade (Figure S4).

The sample preparation was made by the adhesion of the removed elytra piece into a glass microscope plate to make it as plain as possible for further experiments.

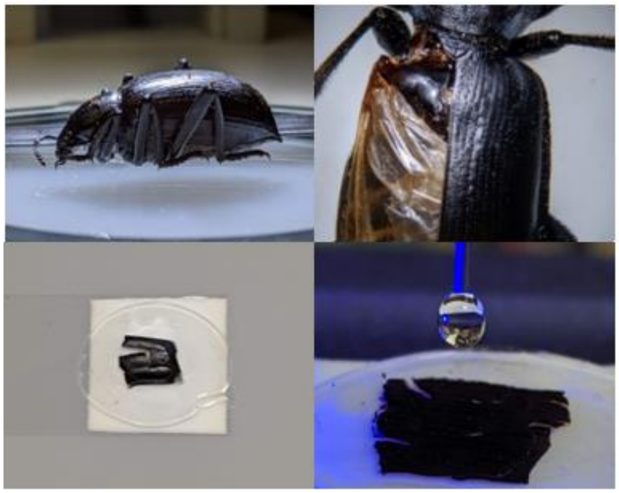

Figure S4. Dissection of the elytra using a surgeon knife and mounting in a microscope slide for analysis.

In Figure S5, we show the micro and nano patterns found by using SEM in every sample of the dissected elytra. It can be described as a honeycomb-like array of polygons along the whole surface area.

Once the morphological features were obtained by SEM images, their hydrophobic properties were tested using an Attension Theta Lite (Biolin Labs) Tensiometer-Goniometer and the static contact angle data was collected in order to compare these results with the ones found in the morphological analysis to correlate both parameters. This data is shown below in Table S2.

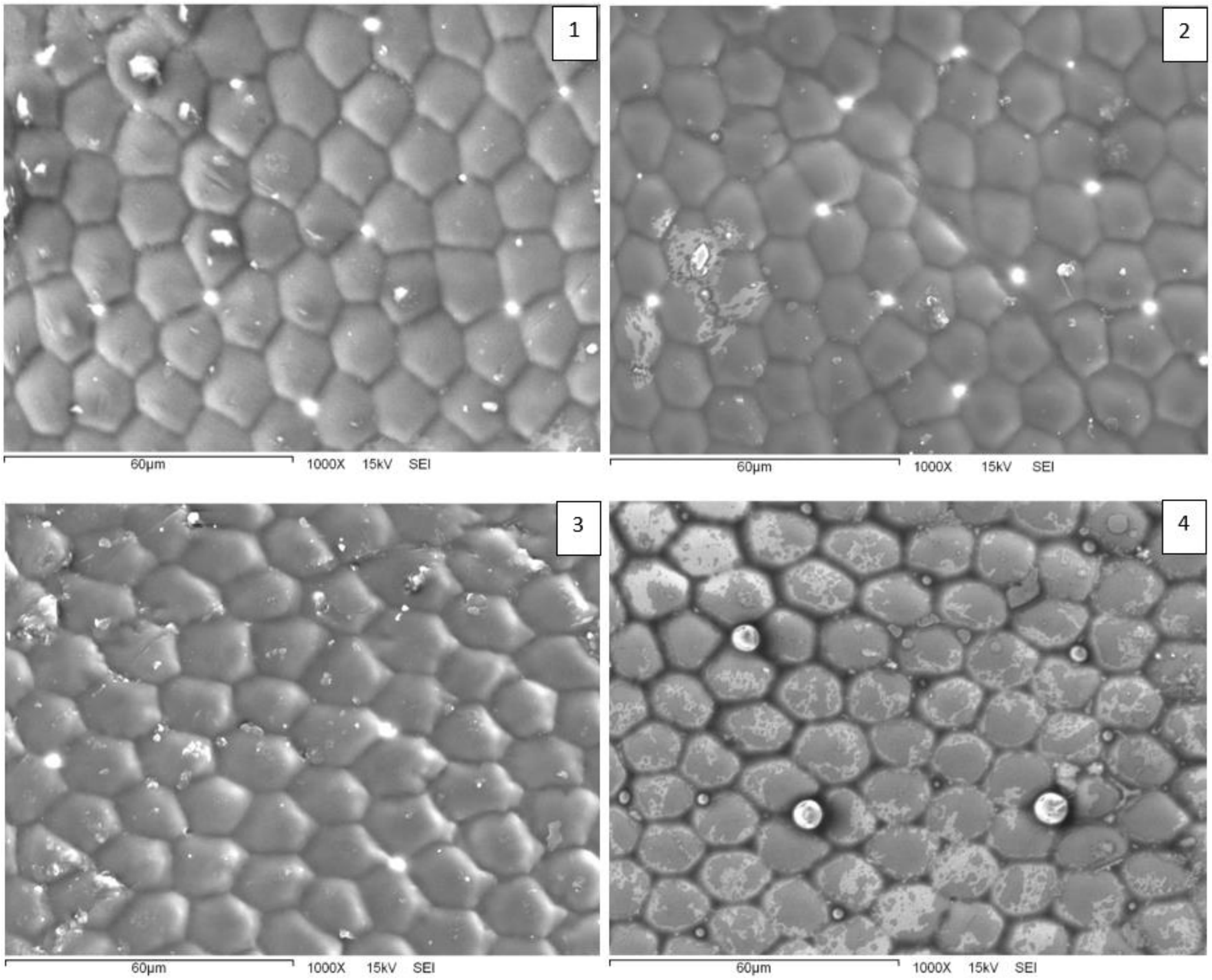

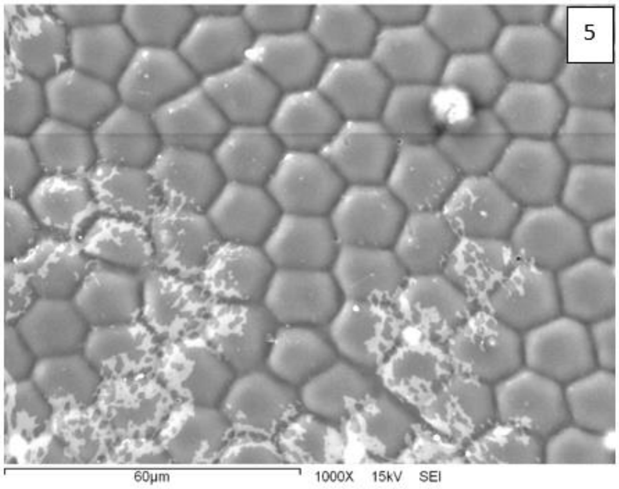
5
60µm
1000X 15kV SEI

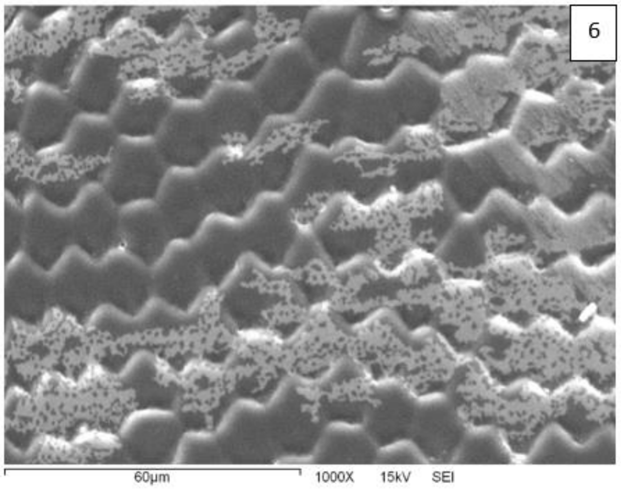
6
60µm
1000X 15kV SEI

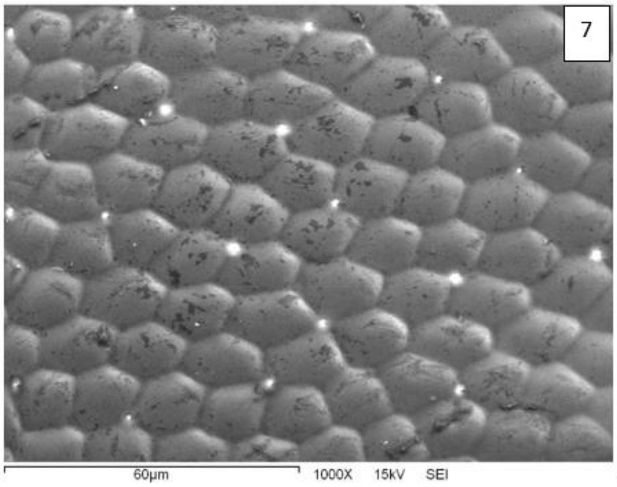
7
60µm
1000X 15kV SEI

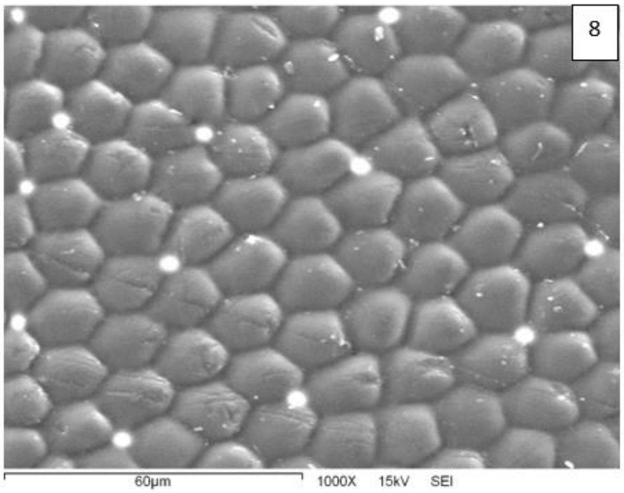
8
60µm
1000X 15kV SEI

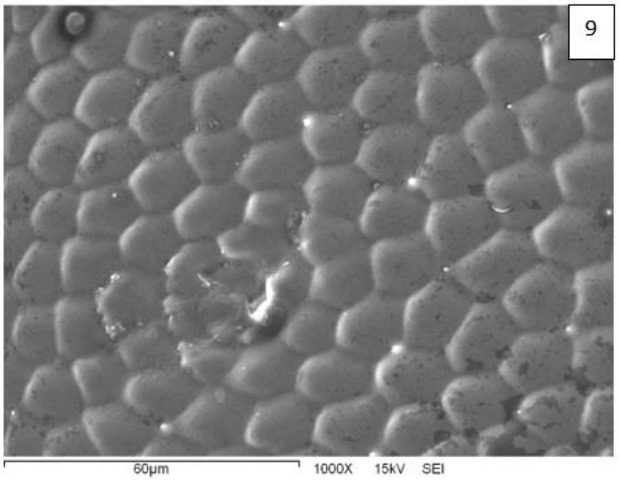
9
60µm
1000X 15kV SEI

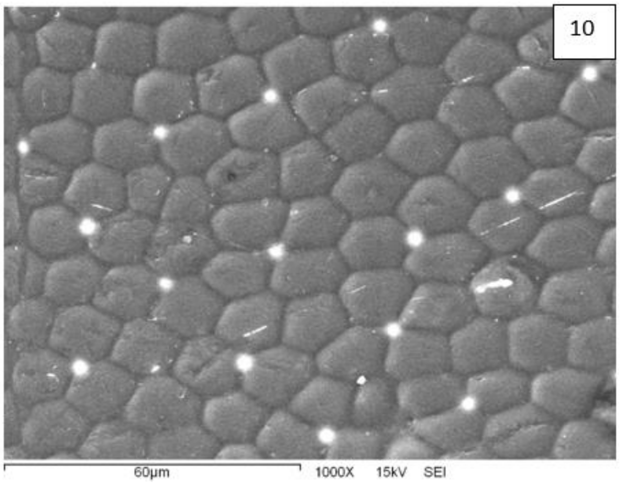
10
60µm
1000X 15kV SEI

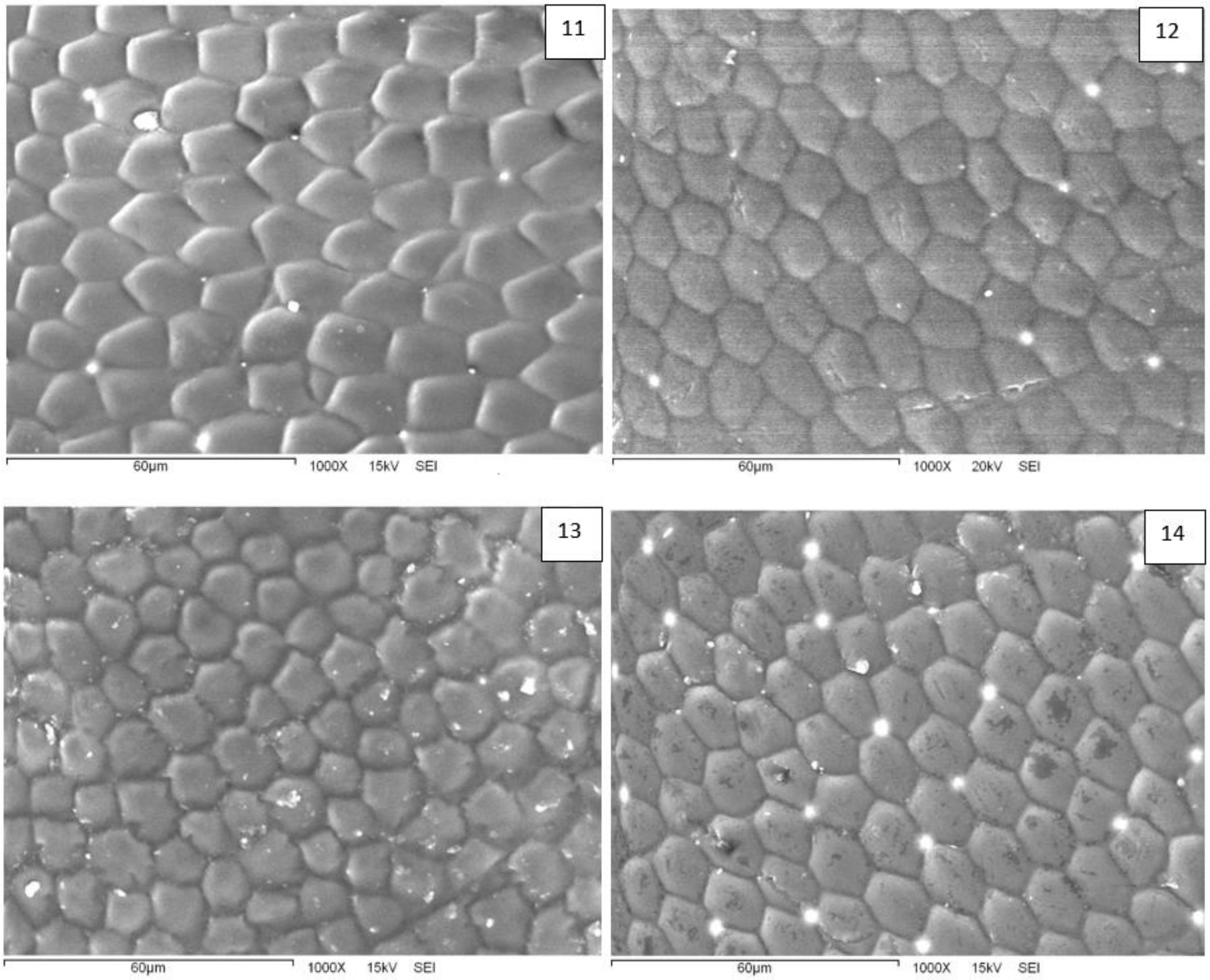

Figure S5. SEM images of *Eleodes eschscholtzii* individuals collected from Hermosillo (1-5) and Bahía Kino (6-14), Sonora, Mexico. Samples at 1000X resolution.

The geometric analysis (Figure S6) was made using Image J (image edition software) to calculate the area of every single polygon and calculating how much of these polygons fitted in a 3600µm2 area to standardize this density of polygons among all the samples (data shown in Table S1).

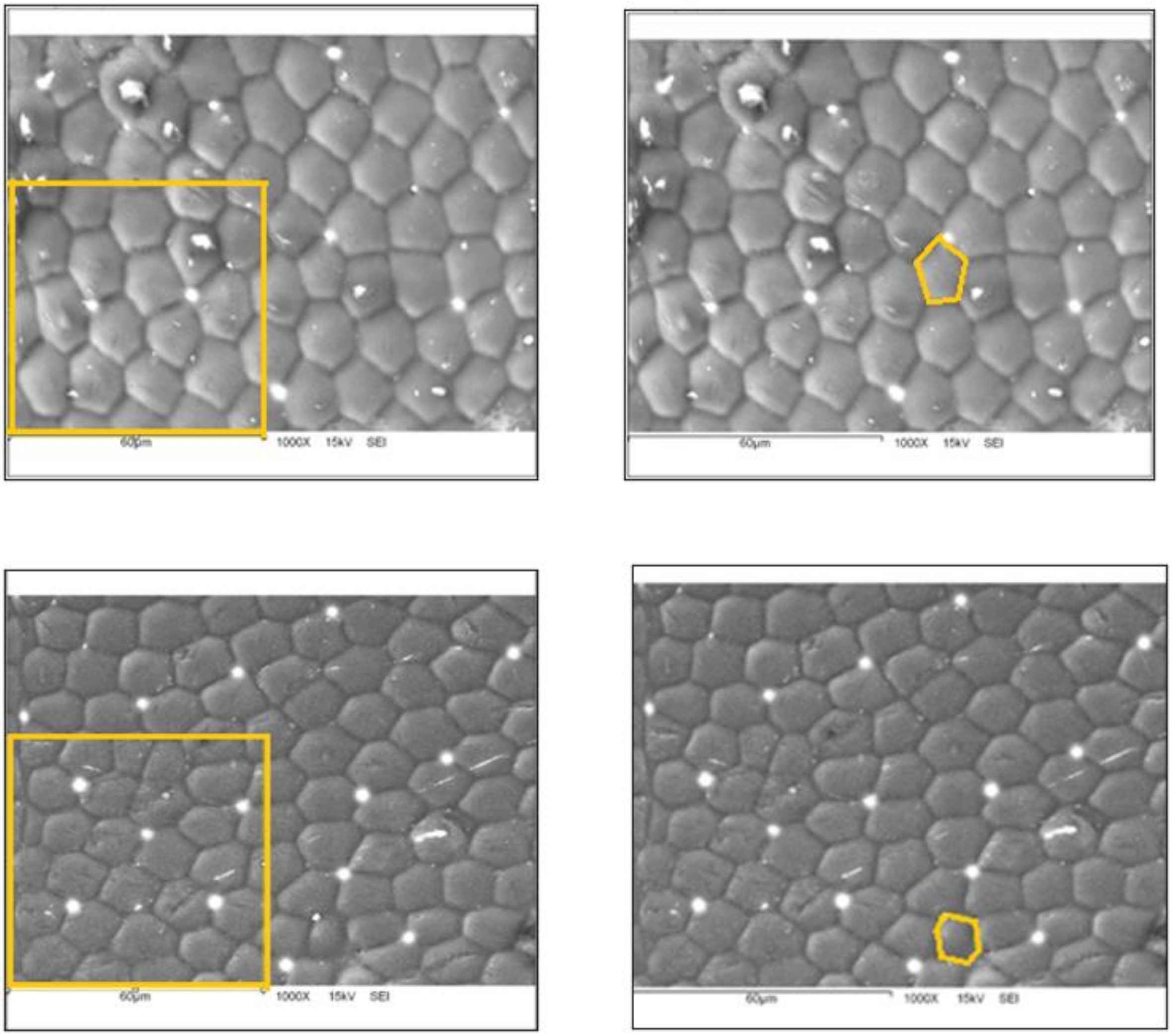

Figure S6. Number of microstructures by area unit of individuals of *Eleodes eschscholtzii* collected from Hermosillo and Bahía Kino, Sonora, Mexico. We used the scale given by the SEM imaging software (60µm = 0.06 mm) to calculate the structural density.

Table S1. Patterns of polygon density in elytra of individuals of *Eleodes eschscholtzii* collected from Hermosillo and Bahía Kino, Sonora, Mexico, from four different samples from both study sites. Samples range in CA values from 52° to 104°. The respective number of polygons per image at 1,000X magnification is also shown. Lower surface areas were recorded for in elytra with the highest CA value for Individual 5 (BK), which corresponds to be the most hydrophobic sample. In contrast, higher surface areas were recorded for individual 1 (HMO), which represents the most hydrophilic sample.

**Site**	**Individual**	**Contact Angle (°)**	**Number of polygons per square unit (3600 μm^2)**	**Polygon surface (μm^2)**
	**1**	52	11.66	308.7
	**2**	60	25.67	140.2
**HMO**	**3**	59	21.85	164.7
	**4**	90	24.31	148.1
	**5**	87	24.77	145.3
	**1**	91	28.91	124.4
	**2**	92	25.1	143.3
	**3**	88	25.82	139.4
	**4**	99	26.89	133.8
**KB**	**5**	104	30.18	119.2
	**6**	96	25.8	139.5
	**7**	84	23.95	150.2
	**8**	99	29.11	123.6
	**9**	75	32.91	109.3

Table S2. Values of contact angle (CA) for individuals captured in Site 1, Hermosillo (HMO) and site 2, Bahía Kino (BK), Sonora, Mexico. Mean and standard deviation for each site is shown. HMO is ca. 100 km away from the coast, BK is coastal.

Individual	Location	Contact angle (°)
1		52 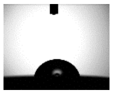
2		60 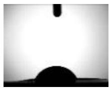
3	**HMO**	59 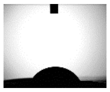
4		90 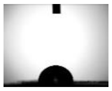
5		87 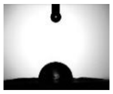
**Average CA value and Standard Deviation for HMO individuals**		**70 ± 18**
1		91 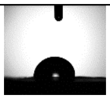
2		92 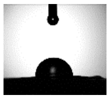
3		88 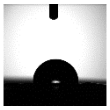
4	**KB**	99 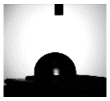
5		104 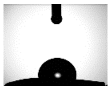
6		96 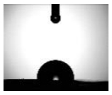
7		84 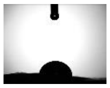
8		99 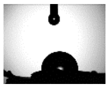
9		75 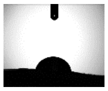
**Average CA value and Standard Deviation for KB individuals**		**92 ± 9**

In Figure S7 we present a FTIR scan for another individual.

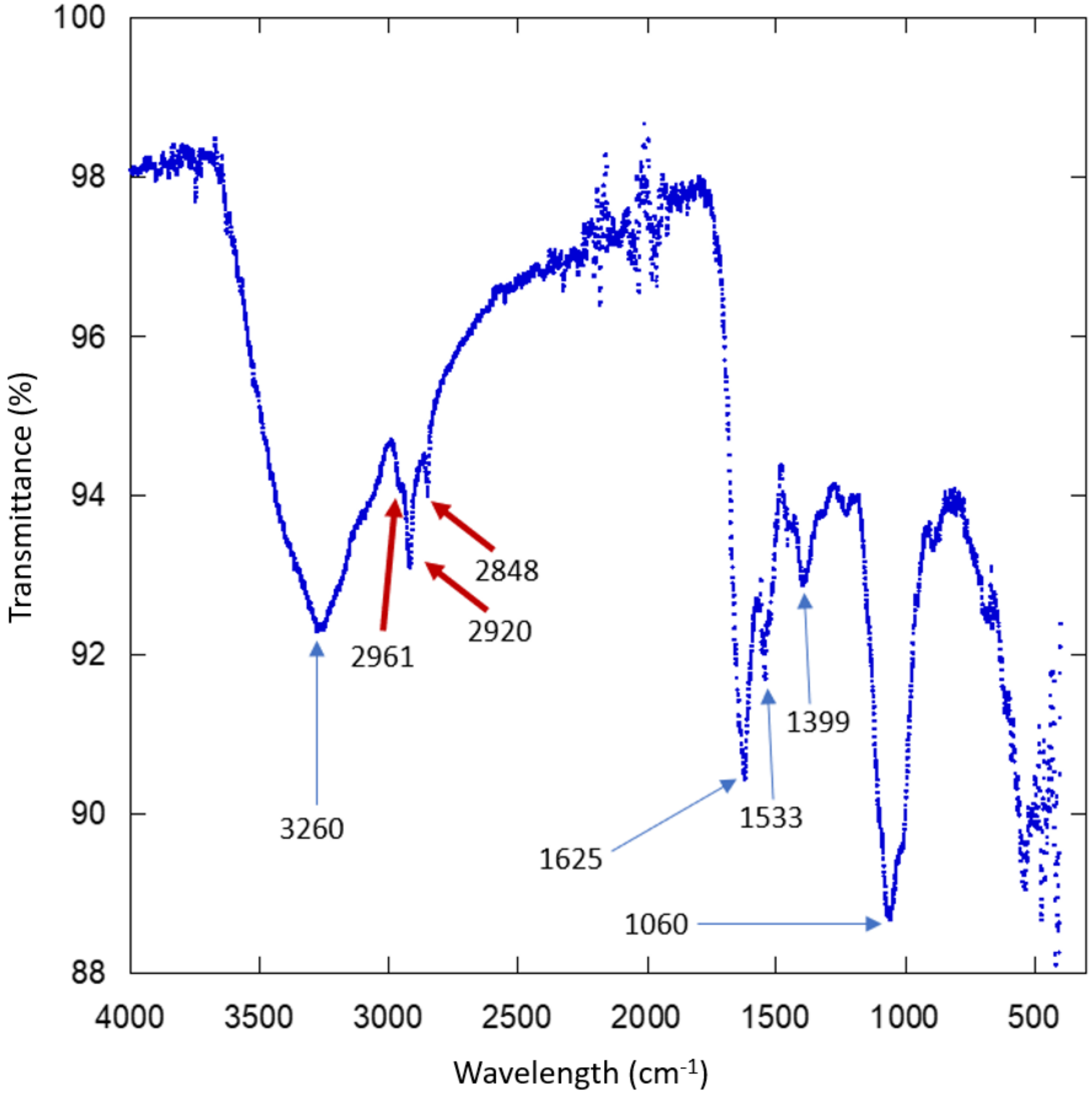

Figure S7: FT/IR scan of elytra from an individual from the KB sampling site.
